# The 26S proteasome drives trinucleotide repeat expansions

**DOI:** 10.1093/nar/gkt295

**Published:** 2013-04-24

**Authors:** Claire Concannon, Robert S. Lahue

**Affiliations:** Centre for Chromosome Biology, School of Natural Sciences, National University of Ireland, Galway, Galway, Ireland

## Abstract

Trinucleotide repeat (TNR) expansion is the causative mutation for at least 17 inherited neurological diseases. An important question in the field is which proteins drive the expansion process. This study reports that the multi-functional protein Sem1 is a novel driver of TNR expansions in budding yeast. Mutants of SEM1 suppress up to 90% of expansions. Subsequent analysis showed that Sem1 facilitates expansions via its function in the 26S proteasome, a highly conserved multi-subunit complex with both proteolytic and non-proteolytic functions. The proteolytic function of the 26S proteasome is relevant to expansions, as mutation of additional proteasome components or treatment of yeast with a proteasome inhibitor suppressed CTG**•**CAG expansions. The 26S proteasome also drives expansions in human cells. In a human astrocytic cell line, siRNA-mediated knockdown of 26S proteasome subunits PSMC5 or PSMB3 reduced expansions. This expansion phenotype, both in yeast and human cells, is dependent on the proteolytic activity of the proteasome rather than a stress response owing to depletion of free ubiquitin. Thus, the 26S proteasome is a novel factor that drives expansions in both yeast and human cells by a mechanism involving protein degradation.

## INTRODUCTION

A group of at least 17 inherited neurological disorders, including Huntington’s disease and myotonic dystrophy type 1, are caused by the same type of genetic mutation: the expansion of trinucleotide repeats (TNRs) (1–4). Among other factors, the likelihood of an expansion depends strongly on the length of the TNR itself. Short TNR tracts are stably transmitted in healthy individuals, whereas longer TNRs are much more prone to expansion. The transition from stable to unstable alleles—the threshold—can occur over a remarkably narrow range of TNR lengths. For example, the threshold in Huntington’s disease falls between 30 and 40 repeats (2,3,5). Expansions that cross into and past this threshold initiate instability and lead to disease. Our laboratory focuses on expansions that occur at or near the threshold. Although these expansions are less common than in long disease-causing alleles, they are key initiating mutations that provoke both high-frequency instability and the onset of symptoms.

Several proteins have been identified that help drive the expansion process. Expansions occur in the presence of these proteins, not their absence, likely because the unusual features of the TNR DNA ‘corrupt’ their normally beneficial biochemical activities (3,6,7). Expansion-promoting factors include certain DNA repair factors, as judged by the suppression of expansions in knockout mice deficient for these repair proteins. Loss of MSH2 or MSH3, the two components of the mismatch repair complex MutSβ, leads to suppression of most inherited and somatic expansions in mice (8–12). Loss of the base excision repair protein NEIL1 suppresses somatic and germ line expansions, particularly in male mice (13). Somatic expansions, but not inherited expansions, are also reduced in animals lacking the mismatch repair factor PMS2, the base excision repair protein OGG1 or the nucleotide excision repair protein XPA (14–16). A second category of expansion-promoting factors includes the histone deacetylase complexes (HDACs) Rpd3L and Hda1 in budding yeast and the human enzymes HDAC3 and HDAC5 (17–19). These HDACs were identified in cell-based expansion assays but have not yet been tested in mice.

The 26S proteasome is a third type of protein factor that facilitates TNR instability. Lin and Wilson (20) showed that treatment of a fibrosarcoma cell line with a proteasome inhibitor, MG132, resulted in a reduced CAG contraction frequency in a transcription-based assay. Thus, when functionally active, the proteasome drives instability in this cell-based assay. The 26S proteasome is a large multi-subunit complex at the core of the ubiquitin-proteasome system (UPS) (21). It is composed of the proteolytic 20S core particle (CP), capped at either end by a 19S regulatory particle (RP) and is highly conserved in evolution. Proteins targeted for degradation by polyubiquitination are brought to the proteasome by ubiquitin shuttle factors. There the proteins are recognized by ubiquitin receptors in the RP, which then acts to unfold, deubiquitinate and translocate the proteins through to the channel of the CP where they are degraded. This degradation role links the proteasome to a large variety of essential cellular functions. However, besides its canonical role in protein degradation, several non-proteolytic roles have also been attributed to the proteasome, mediated by the adenosine triphosphatase (ATPase) subunits of the RP. In DNA repair, the RP has a non-proteolytic role in regulating nucleotide excision repair (NER) by a pathway involving the Rpt6 (Sug1) ATPase and Rad23 shuttle factor (22). In gene expression, the RP can function independently of the activity of the CP to affect histone modification, activator recruitment and stimulation, and transcription elongation (23–26). Thus, when investigating phenotypes associated with disruption of the 26S proteasome, it is important to determine whether the RP and the CP are operating together or independently and to distinguish between proteolytic and non-proteolytic functions.

Independent of the work of Lin and Wilson (20), a genetic screen in *Saccharomyces cerevisiae* revealed that a 26S proteasome subunit, Sem1, promotes expansions (17). The current study investigated the role of the 26S proteasome in TNR expansions. Here, we use genetic and biochemical assays in budding yeast and human cells to show that the 26S proteasome is a molecular driver of expansions, most likely via its proteolytic function.

## MATERIALS AND METHODS

### Yeast strains

BY4741 *sem1*::*kanMX* was purchased from Open Biosystems. All other deletion strains were created via gene deletion cassette in either the BY4741 (*MAT****a*** *his3Δ1 leu2Δ0 met15Δ0 ura3Δ0*) (27) or the S150-2B (*MAT****a*** *leu2-3 leu2-112 his3-Δ trp1-289 ura3-52*) (28) backgrounds. Unless otherwise stated, the S150-2B strain background was used.

### Plasmids

pSEM1 and pDSS1 were gifts from Dr Yahushi Saeki (Tokyo Metropolitan Institute of Medical Science) (29). For both plasmids, the *SEM1* promoter is inserted upstream of the wild-type *SEM1* or *DSS1* genes. pUB is a 2 µ *URA*-marked plasmid that expresses ubiquitin under the control of the CUP1 promoter and was a gift from Prof. Daniel Finley (pUB175, Harvard Medical School).

### Genetic assays and analysis of expanded TNR alleles

The yeast *URA3* and *CAN1* reporter triplet repeat expansion and contraction assays have been described previously (17,30,31). The (CTG)_20_-*CAN1* and (CTG)_25_-*URA3* expansion assays are explained in Supplementary Figure S1A and C for ease of reference. Unless otherwise stated, the (CTG)_20_-*CAN1* reporter assay was used for determining expansion rates. All reporters were integrated into the yeast genome at the *LYS2* locus on chromosome II. The RNA interference experiments and shuttle vector assay in SVG-A cells were performed as described previously (17,18) and are briefly described in Supplementary Figure S5. All siRNAs used were pooled siRNAs purchased from Dharmacon (Scrambled (D-001810), DSS1 (M-021353), PSMC5 (M-009484) and PSMB3 (M-017489). Expansions were verified by single-colony polymerase chain reaction (PCR) across the repeat tract followed by analysis on high-resolution polyacrylamide gels (Supplementary Figure S1B).

### MG132 treatment of yeast

One colony of ∼1 × 10^6^ *erg6* cells with an integrated (CTG)_20_-*CAN1* reporter was split into two 5-ml yeast extract/peptone/dextrose cultures and incubated for 10 doublings with either dimethyl sulfoxide (DMSO) or 100 µM MG132. Deletion of *ERG6* allows MG132 to enter yeast cells (32). An aliquot of each culture was diluted and plated on non-selective plates or plates containing 60 µg/ml of canavanine to measure the expansion frequency. The remaining cultures were lysed, and the whole-cell extracts were analysed by western blot for accumulation of polyubiquitinated proteins as described later in the text.

### Western blot analysis and real-time PCR

Whole-cell lysates were prepared by glass bead disruption (yeast) or by sonication (SVG-A astrocytes). For yeast lysate preparation, logarithmically growing cells were harvested, washed with water and re-suspended in glass bead disruption buffer [50 mM Tris–Cl, pH 7.4, 5 mM ethylenediaminetetraacetic acid (EDTA), 100 mM NaCl, 0.1% Triton X-100, 10% glycerol and 1 mM NaN_3_] with 1 × protease inhibitor cocktail (Sigma). Two hundred microlitres of glass beads was added, and the samples were vortexed at 4°C for 10 min. The samples were microcentrifuged at 4°C at 14 000 rpm for 2 min. The supernatant was retained as whole-cell extract. For SVG-A astrocytes, cells were pelleted and washed twice with ice-cold phosphate-buffered saline (137 mM NaCl, 2.7 mM KCl, 4.3 mM Na_2_HPO_4_ and 1.47 mM KH_2_PO_4_, pH 7.4) before being re-suspended in radioimmunoprecipitation assay buffer (150 mM NaCl, 10 mM Tris–HCl, pH 7.5, 0.1% sodium dodecyl sulphate, 0.1% Triton X, 1% sodium deoxycholate and 5 mM EDTA) with 1 × protease inhibitor cocktail at a concentration of 10^6^ cells/50 µl. Samples were sonicated using a Diagenode Biodisrupter for 5 × 30 s intervals with 30 s on ice in between. Samples were then held on ice for 30 min and microcentrifuged for 40 min at 14 000 rpm at 4°C. The supernatant was retained as whole-cell extract. Protein concentration was determined using the DC assay (BioRad). For detection of protein knockdown after siRNA treatment of SVG-A cells, 50 µg of protein was separated on denaturing polyacrylamide gels. For all experiments involving detection of ubiquitin, 10 µg of yeast or human protein was loaded, and lysis buffers were supplemented with 2 mM iodoacetamide (Sigma) to inhibit cytosolic deubiquitinases. Samples were transferred to a polyvinylidene fluoride membrane. Primary antibodies used were against ubiquitin (sc-8017, Santa Cruz), PSMC5 (NB100-345, Novus Biologicals), PSMB3 (PW8130, Biomol) and β-actin (A2066, Sigma). Secondary antibodies conjugated to horseradish peroxidase were 711-035-152 (anti-rabbit) and 115-035-003 (anti-mouse) from Jackson ImmunoResearch Laboratories. Visualization was by chemiluminescence (Western Lightning Plus-ECL, PerkinElmer). Analysis and quantification was performed using Image J software (rsbweb.nih.gov). Knockdown of DSS1 was quantified by mRNA transcript levels because of previously reported difficulties in performing DSS1 western blot (33–35). RNA was isolated from SVG-A cells using a Qiagen RNeasy Kit, and cDNA synthesis was performed using a Precision nanoScript Reverse Transcription kit (Primer Design, UK). cDNA was analysed using SYBR GreenMaster Mix on the 7500 Fast Real-Time PCR system (Applied Biosystems). Primers used were for DSS1 (forward; CGCGGACAGTCGAGATGTC, reverse; GCCAGCCCAGTCTTCGG) (36) and for HPRT1 (forward; TGACACTGGCAAAACAATGCA, reverse; GGTCCTTTTCACCAGCAAGCT) (37). Using the ΔΔCt method (38), results were normalized for cDNA quantity using HPRT1 control primers, and abundance values were expressed relative to scrambled siRNA, defined as 100%.

### Proteasome activity assay

Approximately 5 × 10^6^ SVG-A cells treated with siRNA, as outlined in Supplementary Figure S5 (17), were resuspended in lysis buffer (13 mM Tris–Cl and 5 mM MgCl_2_, pH 7.8) and subjected to two rounds of freeze–thaw lysis. Lysate was brought up to a final volume of 200 µl by addition of lysis buffer supplemented with 5 mM adenosine triphosphate, 0.5 mM dithiothreitol (DTT), 5 mM EDTA and 100 µM final concentration of fluorescent substrate N-Succ-LLVY-AMC (Chymotrypsin substrate III, Calbiochem, 539142). Fluorescence of released AMC was read at an excitation wavelength of 355 nM, emission wavelength of 460 nm on a Wallac VICTOR3 plate reader. The assay was run for 30 cycles with one measurement per min at 37°C. The rate of activity was calculated from the slope of fluorescence increase over time. Using a standard curve measurement of free AMC (Calbiochem), this slope was calculated as nanomoles AMC released per min. This was then normalized to the protein concentration. Enzyme activity in nanomoles AMC released/min/mg protein used was then normalized to activity in the scrambled siRNA sample.

### Statistical analyses

Yeast expansion rate data are presented in mean centring format. For every experiment, each expansion rate value is normalized to the average wild-type value of that experiment. This allows comparison between experiments where variability in media batches may have altered the absolute expansion rates but not the comparative change between wild-type and mutant strains. The average expansion rate values for each strain are presented in Supplementary Tables S1–S3. For SVG-A cells, summary data are presented in Supplementary Table S4. All *P*-values were determined by two-tailed Student’s *t*-test.

## RESULTS

### Yeast Sem1 promotes CTG•CAG repeat expansions

**Figure 1.**
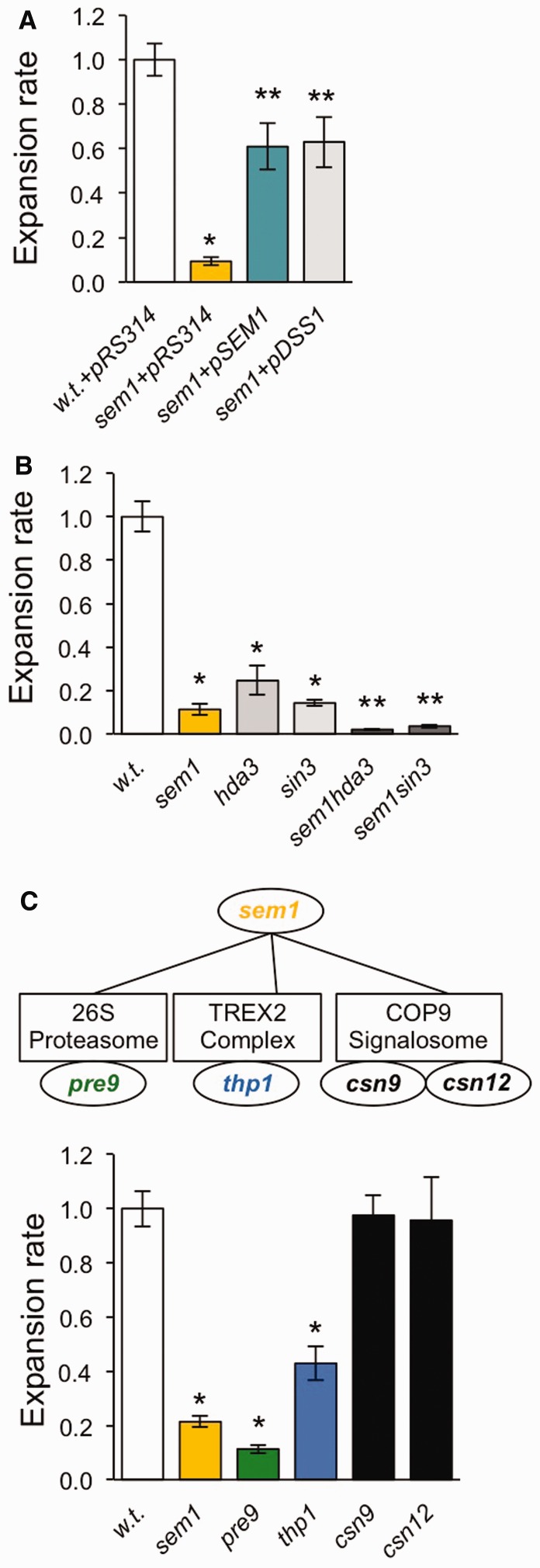
Analysis of expansion rates in *sem1* mutants. For all panels, error bars denote ± SEM. (**A**) Expansion rates in strains that are wild-type (w.t.), *sem1* or *sem1* complemented with a plasmid that expresses either the wild-type *SEM1* gene (pSEM1) or the wild-type human *DSS1* gene (pDSS1) (29). pRS314 is the empty vector control. *P < 0.05, compared with w.t. + pRS314, **P < 0.05 compared with w.t. + pRS314 and with *sem1* + pRS314. (**B**) Genetic interactions between *SEM1* and the histone deacetylase complexes Hda1 (*hda3*) and Rpd3L (*sin3*). *P < 0.05, compared with wild-type, **P < 0.05 compared with wild-type and to each single mutant. As specified in Supplementary Table S1, *P*-values from comparison of single and double mutants ranged from 2.3 × 10^−11^ to 7.7 × 10^−3^. (**C**) Mutational analysis of *SEM1* functions. The *sem1* mutant affects the 26S proteasome, the TREX2 mRNA export complex, the COP9 signalosome and pre-mRNA splicing. These pleiotropic effects were separated using mutants specific to each function, namely, *pre9*, *thp1*, *csn9* and *csn12*, respectively. *P < 0.05, compared with wild-type (*n* and *P* values for all panels are shown in Supplementary Table S1).

If a protein helps drive expansions, then mutation of its corresponding gene should reduce expansion rates. Based on this premise, a screen was performed in *S. cerevisiae* to identify mutants with reduced expansion rates compared with wild-type (17). DNA sequencing identified *SEM1* as a gene whose disruption consistently suppressed expansion rates of a (CTG•CAG)_20_ repeat tract (17). This gene assignment was confirmed in several ways. Targeted disruption of *SEM1* reduced expansion rates to ∼10% of the wild-type level (Figure 1A). This defect was partially rescued by introduction of plasmids expressing either wild-type *SEM1* or its human homologue DSS1 (Figure 1A). The suppressive effect of *sem1* on expansions primarily affects the triplet repeat, not the reporter, because reduced expansion rates were seen for *sem1* mutants using two reporters in two strain backgrounds (Supplementary Figure S1A–D). Additional previously reported phenotypes of *sem1* were also recapitulated in our mutants and were rescued by the plasmids that express wild-type *SEM1* or *DSS1*. Both the targeted *sem1* disruption and the original mutant (M101) were temperature sensitive at 37°C (Supplementary Figure S2A), as previously reported for *sem1* (29,39). Add-back of pSEM1 or pDSS1 reversed this phenotype in the *sem1* strain (Supplementary Figure S2A). Similarly, the accumulation of polyubiquitinated proteins seen in *sem1* mutants (29) could be partially rescued by pSEM1 and pDSS1 (Supplementary Figure S2B). To ensure that the reduced expansion rate phenotype was not an artefact of *sem1* sensitivity to the drugs used to score expansions (5-fluoro-orotic acid (5-FOA) and canavanine), a series of spot tests were performed. Deletion of *SEM1* did not result in increased sensitivity to 5-FOA (Supplementary Figure S3A). *SEM1* deletion was previously reported to confer hypersensitivity to canavanine (39). We confirmed this hypersensitivity to 1 µg/µl of canavanine (Supplementary Figure S3B) and also showed this was dependent on the presence of the *CAN1* gene (Supplementary Figure S3C). To confirm that a *sem1* mutant with an expansion grew normally on canavanine-containing media at the 60 µg/µl concentration used to score expansions, we inserted an expanded (CTG•CAG)_30_ tract into the *CAN1* reporter. There was approximately equal growth for both wild-type and *sem1* strains (Supplementary Figure S3D). We conclude that mutation of *SEM1* suppresses expansions in our system because of effects on the triplet repeat, not on the reporter genes or the drug selections.

As described in Debacker *et al*.** (17), members of the HDACs Hda1 and Rpd3L were also validated as promoting factors of TNR expansions. Components of these HDACs genetically interact with *SEM1* (40–42), and a role for Sem1 in regulating histone modification has been suggested (43,44). We assayed TNR expansions in double mutants of *sem1* with *hda3* (defective in Hda1 complex) or *sin3* (defective in Rpd3L). Single mutants showed similarly reduced levels of expansions to 10–25% of wild-type, but the double mutants *sem1 hda3* and *sem1 sin3* show a much larger effect, reducing triplet repeat expansions to 2–4% of normal rates (Figure 1B). This result indicates that Sem1 promotes expansions by a mechanism that is distinct from these HDACs. Therefore, we addressed alternative functions of Sem1.

The Sem1 protein has several important functions. It is a *bona fide* member of the regulatory subunit of the 26S proteasome in yeast (29,39,45), and this relationship is conserved throughout eukaryotes (34). Genetic interaction maps and subsequent investigations revealed additional roles for yeast Sem1 in pathways of mRNA export, processing and splicing (41,46). To determine which activity of Sem1 is most important for promoting TNR expansions, we tested *SEM1* interacting genes that are specific for each function. Mutations that impair function were included for the proteasome (*pre9*), the TREX2 complex (*thp1*) and the COP9 signalosome (*csn9*) or pre-mRNA splicing (*csn12*). Deletion of *PRE9* resulted in the largest decrease in expansions, to ∼10% of wild-type levels, similar to deletion of *SEM1* (Figure 1C). The *thp1* mutant yielded a more modest reduction, whereas deletion of COP9 signalosome genes *CSN9* and *CSN12* did not affect expansion rates. We conclude that Sem1 facilitates expansions primarily through its proteasome function with a lesser effect mediated through its role in the TREX2 complex.

### TNR expansions are enhanced by the yeast 26S proteasome

**Figure 2.**
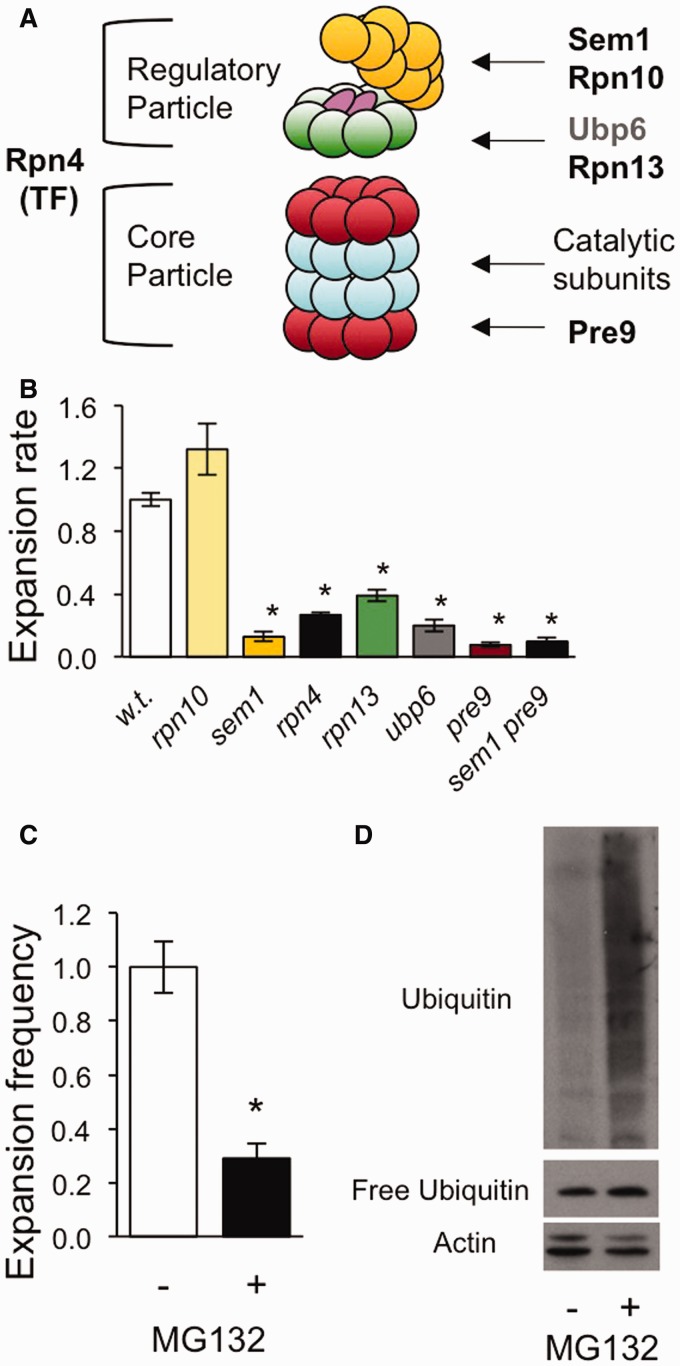
The proteasomal function of Sem1 is important for promoting expansions. (**B** and **C**) Error bars represent ± SEM. (**A**) Schematic of the 26S proteasome divided into the regulatory and core particles, with location of subunits tested. TF = transcription factor. The second regulatory particle, at the ‘bottom’ of the core particle, was omitted for clarity. (B) Effects of deleting proteasome components on expansion rates. *P < 0.05 compared with wild-type. *P-*values from comparison of the *sem1 pre9* double mutant with the single mutants were 0.88 (*sem1*) and 0.50 (*pre9*), (Supplementary Table S1). (C) Decreased expansion frequency on chemical inhibition of the proteasome. Cells were treated with 100 μM MG132 (+) or DMSO only (−) and subsequently tested for expansions. *P < 0.05 compared with DMSO control. (**D**) Representative immunoblot of polyubiquitinated proteins upon MG132 treatment. Cells were treated with DMSO only (−) or MG132 (+) as in C. Cell-free extracts (10 µg protein) were prepared and analysed for polyubiquitinated proteins and free ubiquitin levels using an anti-ubiquitin antibody. Actin was used as a loading control (*n* and *P* values for B and C are shown in Supplementary Table S1).

The similarity between the expansion rate phenotypes of *pre9* and *sem1* strains (Figure 1C) suggested that the 26S proteasome is a promoting factor for TNR expansions. Sem1 is a component of the lid sub-complex of the 19S regulatory particle (Figure 2A). Pre9 is the only non-essential subunit of the 20S core particle in yeast; when deleted, its role is provided by an extra copy of another core component, Pre6 (47). To establish more conclusively whether the proteasome promotes TNR expansions, additional non-essential proteasome subunits or proteasome interacting factors were targeted for deletion (Figure 2A). Aside from *rpn10*, all other five proteasome mutants suppressed expansions (*sem1*, *rpn4*, *rpn13*, *ubp6* and *pre9*; Figure 2B). These genes encode the regulatory particle subunits Sem1 and Rpn13, the proteasome interacting deubiquitinase Ubp6, the core particle subunit Pre9 and the transcription factor Rpn4, involved in expression of the majority of proteasome subunits (21). Moreover, Sem1 and Pre9 promote expansions through a shared pathway, as the double mutant *sem1 pre9* showed an expansion rate phenotype indistinguishable from the two single mutants (Figure 2B), and the spectrum of expansion sizes was similar for both single mutants and the double mutant (Supplementary Figure S4). Proteasome mutants *sem1*, *pre9* and *rpn4*, but not *rpn10*, also showed a reduced expansion rate phenotype when a (CTG•CAG)_25_ *URA3* reporter was used in the BY4741 strain background (Supplementary Figure S1D and Supplementary Table S2), indicating that the expansion phenotype is general for TNR instability and not specific for the *CAN1* reporter. Contraction rates of a (CTG•CAG)_25_ tract were also reduced on deletion of *SEM1* or *PRE9* in a BY4741 strain background (Supplementary Table S2).

Expansions were also suppressed by treatment of proteasome-proficient cells with the proteasome inhibitor MG132. This compound is a reversible inhibitor of the chymotryptic-like activity of the proteasome. In the presence of 100 µM MG132, administered during 10 cell generations, expansions were reduced to ∼30% the level seen in a vehicle-only control (Figure 2C). This finding supports the idea that the proteolytic activity of the proteasome is important for the expansion rate phenotype. Western blotting with an anti-ubiquitin antibody showed the anticipated accumulation of high-molecular weight, polyubiquitinated proteins on MG132 treatment (Figure 2D). Together, the mutant analysis and the inhibitor studies provide compelling evidence that the 26S proteasome is important for promoting TNR expansions in our yeast system.

### The 26S proteasome promotes CTG•CAG expansions in cultured human astrocytes

**Figure 3.**
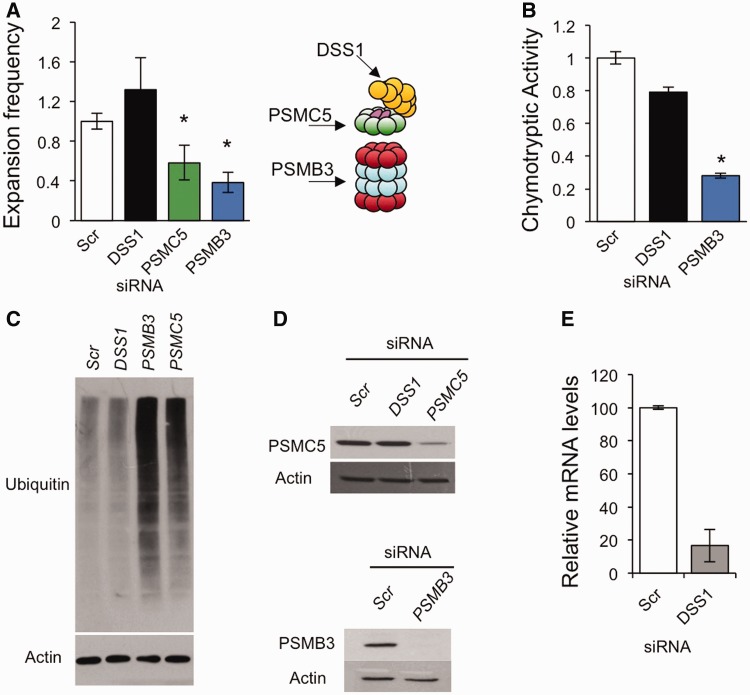
Expansions are suppressed by siRNA knockdown of proteasome components in SVG-A cells. (**A** and **B**) Error bars represent ± SEM. (A) siRNA-mediated knockdown of proteasome subunits PSMC5 and PSMB3 but not DSS1 result in significant decreases in TNR expansion frequencies, *P < 0.05, compared with scrambled (Scr) control. For a summary of the data, see Supplementary Table S4. A schematic of the 26S proteasome shows the location of these subunits. The second regulatory particle, at the ‘bottom’ of the core particle, was omitted for clarity. (B) Cell extracts were prepared after siRNA knockdown and assayed for chymotryptic activity of the proteasome as described in ‘Materials and Methods’ section. *P < 0.05, compared with scrambled control. Scr; *n* = 5, DSS1; *n* = 3, PSMB3; *n* = 4. (**C**) Representative immunoblot of polyubiquitinated proteins. Cells were treated with scrambled control siRNA (Scr) or siRNA to PSMB3, PSMC5 or DSS1, extracts were prepared and 10 µg total protein was analysed by immunoblot for polyubiquitinated proteins (Ubiquitin). Actin was used as a loading control. (**D**) Representative immunoblots for PSMC5 and PSMB3 knockdown. Fifty micrograms of total protein was loaded in each lane. Actin was used as a loading control. Additional knockdown data are presented in Supplementary Figure S6. (**E**) Knockdown of DSS1 as measured by mRNA level, as described in ‘Materials and Methods’ section. Error bar denotes ± SEM, *n* = 3.

The 26S proteasome is highly conserved from yeast to humans. To address whether the role of the proteasome in promoting TNR expansions is also conserved, human proteasome subunits were targeted by siRNA-mediated knockdown. We used SVG-A cells, an immortalized cell line derived from human astrocytes (48,49), because this line supports expansions in culture (17,18,50). The scheme for knockdown and assay of functional outcomes is presented in Supplementary Figure S5. The targets for knockdown were DSS1, PSMC5 and PSMB3 (Figure 3A). DSS1 is the human homologue of yeast Sem1 and also a member of the 19S regulatory particle (34,45). However, yeast and human proteasomes show different dependency on the two proteins. Yeast mutants lacking *SEM1* are defective for proteasome activity (29,39), whereas knockdown of DSS1 in human cells results in only slight effects on proteasome function (34,36,51), perhaps because of genetic redundancy. We confirmed these observations by showing that yeast *sem1* mutants accumulate polyubiquitinated proteins (Supplementary Figures S2B and S4B), but knockdown of DSS1 results in only a slight effect on proteasome activity (Figure 3B and C). PSMC5 (also known as SUG1, p45 and/or TRIP1) is an ATPase subunit in the base of the regulatory particle homologous to the essential yeast subunit Rpt6 (52,53). PSMB3 is a non-proteolytic β-type subunit of the core particle (54). PSMC5 and PSMB3 were chosen for knockdown to target the regulatory and core particles, respectively, and their successful knockdown by siRNA has been previously reported (51,55,56).

There was significant suppression of TNR expansion frequencies upon knockdown of the regulatory particle subunit PSMC5 or the core particle subunit PSMB3, but not DSS1 (Figure 3A). Expansions were reduced to 58% or 38% of control levels upon treatment of the cells with siRNA targeting PSMC5 or PSMB3, respectively. The effects of DSS1 or PSMB3 knockdown on proteasome activity (Figure 3B) paralleled the expansion results. As expected, DSS1 knockdown showed little effect on biochemical assays for the chymotryptic function of the proteasome, whereas PSMB3 knockdown reduced activity to 28% of control levels. Ablation of PSMC5 and PSMB3 also led to the expected accumulation of polyubiquitinated proteins (Figure 3C), indicative of a proteolytic defect. This accumulation was not seen upon DSS1 knockdown.

The knockdown efficiencies for PSMC5 and PSMB3 were to final levels of 33% and 17%, respectively, as measured by immunoblot (Figure 3D and Supplementary Figure S6). Knockdown of DSS1 was also efficient, to 17%, as measured by transcript level (Figure 3E). The spectra of expansion sizes were similar in control cells as in all three knockdown experiments (Supplementary Figure S7). From a starting tract of 22 repeats, 4–17 additional repeats were added to give final allele sizes of 26–39 repeats. Thus, a number of expansions in this system cross into the crucial threshold of 30–40 repeats where instability becomes prominent in humans and disease can initiate (2,3,5). We conclude that the human 26S proteasome enhances expansions of threshold-length triplet repeats in SVG-A cells.

### Depletion of ubiquitin levels cannot explain the triplet repeat expansion phenotype

**Figure 4.**
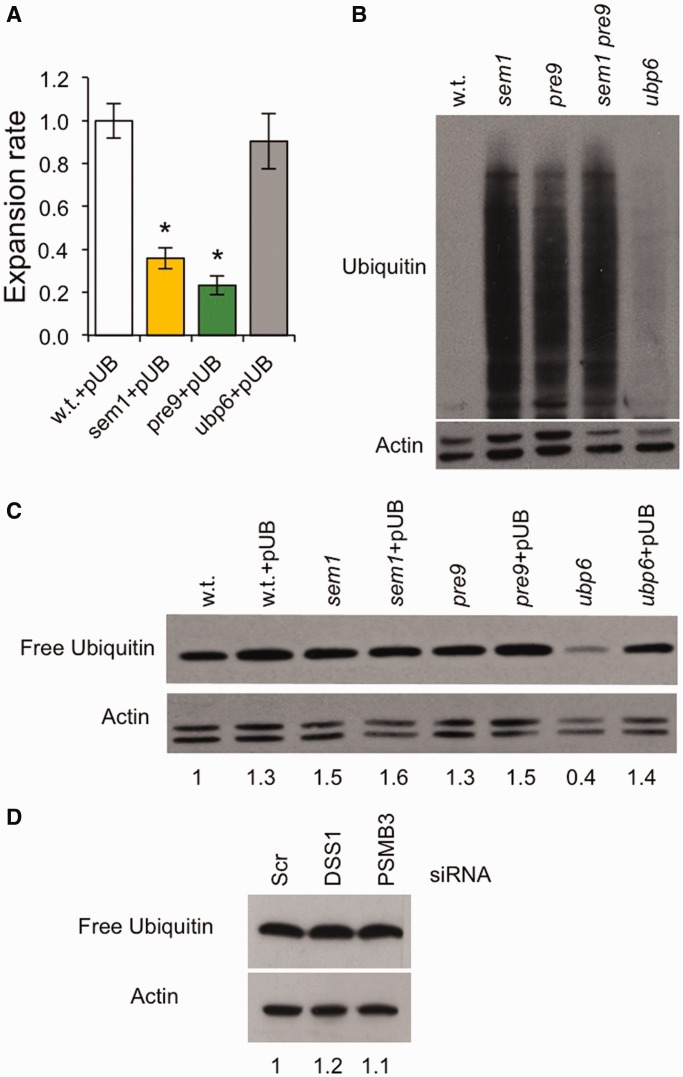
Expansion differences cannot be explained by changes to free ubiquitin levels. (**A**) Expansion rates in strains that are wild-type, *sem1*, *pre9* or *ubp6* containing the pUB plasmid that expresses wild-type levels of ubiquitin (57). Error bars represent ± SEM. *P < 0.05, compared with w.t. control. (**B**) Western blot analysis of polyubiquitinated proteins in proteasome mutants *sem1*, *pre9*, *sem1 pre9* and *ubp6*. (**C**) Analysis of free ubiquitin levels in yeast strains with or without pUB plasmid. The numbers show the relative levels of free ubiquitin compared with wild-type without pUB (w.t.) levels after normalization to actin. (**D**) Immunoblots of SVG-A extracts after treatment with scrambled (Scr) siRNA or siRNA directed against DSS1 or PSMB3. For B–D, 10 µg total protein was loaded in each lane, and actin was used as a loading control.

For both yeast and human cells, we investigated the possibility that proteasome deficiency or inhibition could deplete the levels of free ubiquitin, leading to a stress response that indirectly suppressed expansions. If so, a prediction is that add-back of ubiquitin on a yeast plasmid would overcome the expansion deficit in *sem1* or *pre9* cells. To do this, we made use of a plasmid that contains the ubiquitin gene under the control of a copper inducible promoter. In the absence of copper, this plasmid expresses ubiquitin at approximately wild-type levels (57). The data show a continued expansion deficit in *sem1* and *pre9* strains harbouring the ubiquitin-expressing plasmid pUB (Figure 4A). Thus, depletion of ubiquitin levels is not responsible for the reduced expansion rate phenotype in mutants with impaired proteolytic activity. We confirmed this conclusion through the use of an *ubp6* mutant. In the absence of the deubiquitinase Ubp6, ubiquitin is taken into the core particle and degraded, leading to depletion of ubiquitin levels without affecting the activity of the proteasome (57–59). Proteasome activity was assessed by examining levels of polyubiquitinated proteins (Figure 4B). Polyubiquitinated proteins did accumulate in proteasome-defective mutants *sem1*, *pre9* and *sem1 pre9* but not in the *ubp6* strain (Figure 4B). In the expansion assay, add-back of the pUB plasmid to the *ubp6* strain rescued the expansion rate phenotype back to wild-type levels, unlike the continued expansion deficit in *sem1* and *pre9* mutants (Figure 4A). Thus, it is disruption of proteasome activity, and not depletion of ubiquitin levels, that is responsible for the decrease in TNR expansion rates in *sem1* and *pre9* mutants.

A second prediction of the indirect stress response theory is that free ubiquitin levels should be detectably reduced in yeast proteasome mutants. As seen in Figure 4C, this is not the case for *sem1* and *pre9* mutants, which show similar free ubiquitin levels to wild-type strains. These levels increased slightly when pUB was present. There was also no apparent depletion of free ubiquitin when wild-type cells were treated with MG132 (Figure 2D). As described previously (57), free ubiquitin levels did decrease in *ubp6* strains and were restored on add-back of the pUB plasmid (Figure 4C). In SVG-A cells, knockdown of PSMB3, but not DSS1, results in impaired proteolytic activity (Figures 3B and C). We found that free ubiquitin levels were indistinguishable between DSS1 or PSMB3 knockdown cells compared with control siRNA cells (Figure 4D). As in yeast, these data in human cells do not support an indirect stress response as the major mechanism for suppressing expansions. We conclude, instead, that expansions arise from a more direct role of the proteasome requiring its proteolytic function.

## DISCUSSION

This study reveals a novel role for the 26S proteasome in driving expansions of threshold-length TNRs in both budding yeast and human astrocytes. A blind screen for promoting factors of TNR expansions in *S. cerevisiae* identified the *SEM1* gene (17). Subsequent analysis showed that CTG•CAG expansions and contractions are stabilized in *sem1* mutants. By genetic analysis, we determined that the proteasomal function of Sem1 is important for promoting TNR instability. Another function of Sem1 in the TREX2 mRNA export complex seems to play a lesser role in expansions; however, this was not pursued further. Interfering with 26S proteasome function through mutation or chemical inhibition in yeast suppressed TNR expansions. This role of the 26S proteasome is conserved in human cells, as siRNA-mediated knockdown of proteasome subunits in a human astrocytic cell line also suppressed expansions in a manner that coincided with loss of proteolytic activity. Our data exclude an indirect effect on expansions through a stress response triggered by changes in free ubiquitin levels. Previously, treatment of human fibrosarcoma cell line with MG132 resulted in significant decrease in the frequency of transcription-induced CAG contractions (20). We show here that the proteasome also affects CTG•CAG expansions in both yeast and human cells.

Deletion of several yeast proteasome subunits (*sem1*, *rpn13* and *pre9*) resulted in a decrease in TNR expansion rates. In addition, an expansion defect was also seen upon loss of the transcription factor Rpn4, which controls the expression of the majority of the proteasome genes, and whose deletion results in decreased levels of proteasome activity (60). Thus, in most cases, mutations that affect proteasome subunits led to suppression of TNR expansion rates. In contrast, expansions were unchanged in a strain missing Rpn10, a ubiquitin receptor that binds to polyubiquitinated proteins targeted for degradation (61,62). It is possible that the lack of expansion rate phenotype in an *rpn10* mutant is because the subset of proteins it recognizes does not include those involved in the TNR expansion phenotype. For example, Elangovan *et al*.** (63) described different substrate specificity between ubiquitin receptors in HeLa cells that are homologous to yeast Rpn10 and Rpn13. We also note that the expansion-suppressing phenotype of the *ubp6* mutant is most likely indirect. Although not a proteasome subunit per se, Ubp6 deubiquitinase activity is dependent on association with the base of the RP (58), and Ubp6 levels at the proteasome can be altered in response to ubiquitin levels (57). We found that, in a *ubp6* mutant, addition of a ubiquitin-expressing plasmid resulted in expansion rates and free ubiquitin levels returning to normal. Thus, the expansion rate suppression in *ubp6* cells is likely because of indirect effects of ubiquitin signalling. This finding is distinct, however, from *sem1* and *pre9* mutants, where expansion defects and free ubiquitin levels were not much altered in the presence of the ubiquitin-expressing plasmid. Overall, the data support the proteasome as a driver of TNR expansions in yeast.

Evidence in both yeast and human cells implicate the proteolytic function of the proteasome as important for TNR expansions. Sem1 deletion results in proteasome instability and a proteolytic defect (29,39). As an α-type subunit Pre9 acts to gate the CP channel, and deletion of *PRE9* results in reduced levels of mature proteasomes (47). Deletion of *SEM1* and *PRE9* resulted in accumulation of polyubiquitinated proteins in our strains, indicative of a defect in proteolysis. A double *sem1 pre9* mutant suppressed expansions to the same extent of either of the single mutants. These genetic data indicate that in yeast, both the RP and CP work together to drive expansions, and that the proteolytic activity of the proteasome is important. These mutant effects can be phenocopied in wild-type cells by addition of MG132, which inhibits the chymotryptic activity of the proteasome. Proteolytic activity was also found to be crucial for expansions in human cells. In the SVG-A astrocytic cell line, expansions were suppressed on knockdown of the RP subunit, PSMC5, or the CP subunit, PSMB3. In contrast, knockdown of the Sem1 human homologue, DSS1, did not reduce the occurrence of expansions. These effects on expansion frequencies mirror the levels of proteolytic activity after knockdown. Previous investigations also found a significant reduction in proteasome activity upon siRNA-mediated depletion of PSMB3 (51) but not DSS1 (34,36,51). Although PSMC5 is primarily studied for its non-proteolytic roles in transcription, knockdown of PSMC5 also affects the proteolytic activity of the proteasome (55,64). It has been suggested that DSS1 has two functions in human cells, with a primary role in stabilizing BRCA2 protein and a secondary role as a proteasome subunit (33,65,66). Thus, proteasome function is significantly more dependent on Sem1 in yeast than on DSS1 in human cells. In summary, only those 26S proteasome subunit knockdowns that strongly affect proteolytic activity, PSMC5 and PSMB3, result in decreased expansion frequency in SVG-A cells.

How might the proteasome be affecting TNR expansions? Epistasis analysis of *sem1* and mutants in HDAC components show that the proteasome and HDACs affect TNR expansion by distinct mechanisms. The proteasome may be degrading protecting factors that otherwise would act to resolve the abnormal secondary structures formed by TNRs without causing expansion. DNA repair is an important mechanism by which triplet repeat abnormal secondary structures can be converted to expansions (3,6,7). Increasing evidence links the proteasome to important processes during the DNA damage response and repair pathways. The proteasome is required for degradation of certain DNA repair proteins, is important for downstream signalling and has been shown to be recruited to DNA double-strand breaks (45,67,68). Thus, the proteasome, either by indirect proteolysis or by degrading factors at the TNR itself, could negatively alter repair pathways required to correctly resolve TNR DNA.

Other groups have suggested that RNA polymerase II (RNA pol II) stalls near TNRs and is targeted by the mammalian proteasome. Lin and Wilson (20) suggested that triplet repeat instability could be mediated by proteasomal degradation of stalled RNA Pol II at R-loops, which is the damage recognition step for activating the transcription-coupled NER (TC-NER) pathway. This group also showed that siRNA-mediated knockdown of the elongation factor TFIIS reduced CAG contraction frequencies (20). Several additional studies strengthened the correlation between R-loop formation and TNR instability (69–71), although we are not aware in these reports of a direct link to the proteasome. A recent article found a correlation between RNA Pol II levels and CAG instability in a Huntington’s disease mouse model (72). Specifically this study found that the presence at the HD gene of the elongating Ser2 phosphorylated form, but not the initiating Ser5 phosphorylated form, corresponded with CAG instability. Ubiquitination and degradation of RNA Pol II are specific for this elongating form (73). One possibility is that 26S proteasome-mediated degradation of elongation form RNA Pol II stalled at TNR DNA could initiate a TC-NER pathway that results in expansions. In an XPA-deficient spinocerebellar ataxia (SCA1) mouse model, (CAG) repeats were stabilized in certain neuronal cell types (16), and XPA can bind to hairpin DNA *in vitro* (74). The proteasome has established roles in controlling NER activity both through proteolytic and non-proteolytic activities (22). Thus, it is possible that the proteasome is coordinating RNA Pol II degradation and NER activity to enhance TNR instability.

Currently the proteasome inhibitor bortezomib is being used for treatment of haematological malignancies. Could proteasome inhibitors play a role in slowing the progression of somatic expansions in TNR disorders? The proteasome is important for the clearance of toxic polyglutamine protein aggregates, which result from expression of an expanded (CAG) tract in the protein coding region of a gene such as in Huntington’s disease (HD) or spinocerebellar ataxia type 1 (75). Thus, inhibiting proteasome activity would not be beneficial in such polyglutamine disorders, and proteasome impairment in the striatum of HD mice worsens the disease (76). However, the use of proteasome inhibitors in the non-polyglutamine disorders, such as myotonic dystrophy type 1 and Friedreich’s ataxia, could potentially suppress somatic expansions without having an adverse effect on the disease pathology. Proteasome inhibition affects a wide range of cellular pathways, and focus is now switching to more precise inhibition of the UPS through targeting of the ubiquitin pathway enzymes (77). For example, inhibition of a specific E3 ubiquitin ligase that acts at a non-proteasomal site allows greater specificity and less off-target effects. Thus, identification of the proteasome targets that are responsible for promoting TNR instability could lead to development of target-specific modifiers. Stabilization of these factors could, therefore, help reduce instability without adversely affecting the activity of the proteasome.

## SUPPLEMENTARY DATA

Supplementary Data are available at NAR Online: Supplementary Tables 1–4 and Supplementary Figures 1–7.

## FUNDING

Science Foundation Ireland [10/IN.1/B2973 to R.S.L.]; Thomas Crawford Hayes Fund (to C.C.); College of Science, National University of Ireland, Galway (to C.C.). Funding for open access charge: Science Foundation Ireland [10/IN.1/B2973].

*Conflict of interest statement*. None declared.

## Supplementary Material

Supplementary Data
