# Supplementary material for: The 26S proteasome drives trinucleotide repeat expansions
Source: Nucleic Acids Res. 2013 Apr 24;41(12):6098–108. doi: 10.1093/nar/gkt295 (PMC3695522; doi:10.1093/nar/gkt295)
Supplement: Supplementary Data [file supp_41_12_6098__index.html]

The 26S proteasome drives trinucleotide repeat expansions — The 26S proteasome drives trinucleotide repeat expansions — Supplementary Data 

# The 26S proteasome drives trinucleotide repeat expansions

## Supplementary Data

files

**Files in this Data Supplement:**

- Supplementary Data - pdf file
