# Supplementary material for: The 26S proteasome drives trinucleotide repeat expansions
Source: Nucleic Acids Res. 2013 Apr 24;41(12):6098–108. doi: 10.1093/nar/gkt295 (PMC3695522; doi:10.1093/nar/gkt295)
Supplement: Supplementary Data [file supp_gkt295_nar-00557-f-2013_-File006.pdf]

Supplementary Information

The 26S proteasome drives trinucleotide repeat expansions

Claire Concannon and Robert S. Lahue*

Centre for Chromosome Biology

School of Natural Sciences

National University of Ireland, Galway

Galway, Ireland

Supplementary tables 1-4; Supplementary figure legends and figures 1-7

Supplementary Table 1

Expansions of (CTG)$_{20}$ CAN1 reporter in yeast S150-2B background

Figure 1A genotype	*n*	fold change	SEM	*P* vs w.t.	*P* vs *sem1*
w.t.+pRS314	11	1	0.07	-	2.47E-09
sem1+pRS314	9	0.09	0.02	2.47E-09	-
sem1+pSEM1	11	0.61	0.10	6.46E-03	5.07E-04
sem1+pDSS1	11	0.63	0.11	1.29E-02	7.84E-04

(average wild type rate = 2x10^{-3})

Figure 1B genotype	*n*	fold change	SEM	*P* vs w.t.	*P* vs *sem1*	*P* vs *hda3*	*P* vs *sin3*
w.t.	17	1	0.07	-			
sem1	17	0.11	0.02	1.49E-10			
hda3	6	0.25	0.07	7.15E-07			
sin3	6	0.14	0.01	9.16E-10			
sem1hda3	10	0.02	0.004	2.03E-10	1.96E-03	2.27E-11	
sem1sin3	9	0.04	0.01	1.97E-10	7.71E-03		1.49E-07

(average wild type rate = 3x10^{-4})

Figure 1C genotype	*n*	fold change	SEM	*P* vs w.t.
w.t.	17	1	0.07	-
sem1	17	0.21	0.02	8.50E-10
pre9	12	0.11	0.02	2.28E-10
thp1	5	0.97	0.08	1.38E-05
csn9	10	0.43	0.06	8.14E-01
csn12	7	1.57	0.26	8.16E-01

(average wild type rate = 5x10^{-5})

Figure 2B genotype	*n*	fold change	SEM	*P* vs w.t.	*P* vs *sem1*	*P* vs *pre9*
w.t.	23	1	0.04	-		
sem1	20	0.13	0.03	9.09E-19		
pre9	18	0.07	0.02	1.33E-19		
rpn10	7	1.32	0.16	1.10E-01		
rpn13	10	0.39	0.04	2.24E-10		
rpn4	9	0.27	0.02	4.26E-12		
ubp6	13	0.20	0.04	7.05E-15		
sem1 pre9	23	0.10	0.03	3.49E-21	8.84E-01	4.98E-01

(average wild type rate = 3x10^{-4})

Figure 2C genotype	n	fold change	SEM	p vs DMSO
DMSO	6	1	0.09	-
MG132	6	0.29	0.06	1.98E-04

(average DMSO expansion frequency = 3x10^{-3} over 10 generations)

Figure 4A genotype	n	fold change	SEM	p vs w.t.+pUB
w.t.+pUB	33	1	0.08	-
sem1+pUB	25	0.36	0.05	7.43E-08
pre9+pUB	18	0.23	0.04	2.35E-08
ubp6+pUB	10	0.90	0.13	5.70E-01

(average wild type rate = 1x10^{-4})

Supplementary Table 2

Expansions & contractions in yeast BY4741 background

A

Expansions of $(CTG)_{25}$ - URA3 Reporter

genotype	n	fold change	SEM	p vs w.t.
w.t.	11	1	0.11	-
sem1	7	0.36	0.05	1.26E-04
pre9	5	0.36	0.07	2.59E-04
rpn4	4	0.05	0.02	2.20E-04
rpn10	4	1.20	0.09	4.78E-01

(average wild type rate $1x10^{-4}$)

B

Contractions of $(CTG)_{25}$ + $(C,T,G)_8$ - URA3 Reporter

genotype	n	fold change	SEM	p vs w.t.
w.t.	9	1	0.06	
sem1	11	0.22	0.03	1.33E-07
pre9	14	0.36	0.02	1.67E-06

(average wild type rate $4x10^{-4}$)

Supplementary Table 3

Expansion rate data for sem1 mutants

Expansions of $(CTG)_{20}$ - CAN1 Reporter in S150-2B background

genotype	n	fold change	SEM	p vs w.t.
w.t.	28	1	7.61E-06	-
sem1	30	0.18	1.24E-06	1.04E-09

(average wild type rate $1x10^{-4}$)

Expansions of $(CTG)_{20}$ - CAN1 Reporter in BY4741 background

genotype	n	fold change	SEM	p vs w.t.
w.t.	28	1	7.61E-06	-
sem1	30	0.18	1.24E-06	1.04E-09

(average wild type rate $5x10^{-5}$)

Expansions of $(CTG)_{25}$ - URA3 Reporter in S150-2B background

genotype	n	fold change	SEM	p vs w.t.
w.t.	3	1	5.57E-06	-
sem1	8	0.28	3.40E-06	5.28E-03

(average wild type rate $6x10^{-5}$)

Expansions of $(CTG)_{25}$ - URA3 Reporter in BY4741 background

genotype	n	fold change	SEM	p vs w.t.
w.t.	11	1	0.11	-
sem1	7	0.36	0.05	1.26E-04

(average wild type rate $1x10^{-4}$)

Supplementary Table 4

Expansion frequency data for SVG-A siRNA experiments

siRNA	Representative samples			Summary Data		Background
	Expansion Frequency (per 10^5 events)	Fraction of confirmed events	Corrected expansion frequency (per 105 events)	Number of repetitions (*n*)	Fraction of Scr siRNA expansion frequency (Mean ± SEM)	Corrected background expansion freq./Scr expansion freq.
Scr	430	6/20	129	8	1 (± 0.08)	
DSS1	1630	3/19	257	3	1.32 (± 0.32)	
PSMC5	267	6/20	80	5	0.58 (± 0.17)	0.05
PSMB3	149	6/19	47	3	0.38 (± 0.10)	

Supplementary Figure 1. Genetic assays used to monitor TNR expansions in yeast. (**A**) Yeast *CAN1* TNR expansion reporter assay. At the starting TNR length of $(CTG)_{20}$ the *CAN1* gene is expressed and the yeast die on media containing the drug canavanine. Expansions of ≥ 6 repeats alters the space between the TATA box and the preferred site of transcription initiation, resulting in incorporation of an out-of-frame ATG codon that blocks expression of the *CAN1* reporter gene. Drug resistance ensues. Unless otherwise specified this is the reporter used. (**B**) Expansions are verified by PCR across the TNR tract with Cy-5 labeled primers and subsequent analysis on a high-resolution poly-acrylamide gel (30). This allows adjustment of TNR expansion rates for the percentage of real expansions. Shown are 20, 22 and 25 repeat standards, starting tracts of 20 repeats and PCR products of canavanine resistant (Can^R) colonies showing expansion of the starting TNR. (**C**) Yeast *URA3* TNR expansion reporter assay. This operates on the same premise as the *CAN1* reporter. The differences are that the starting tract is $(CTG)_{25}$ and expansions of ≥ 5 repeats are required to block expression of the *URA3* gene. Colonies with expansions are scored by resistance to 5-FOA. (**D**) Expansion rates of *sem1* mutants in two different strains (S; S150-2B background, B; BY4741 background) with two different reporters [C; $(CTG)_{20}$-*CAN1*, U; $(CTG)_{25}$-*URA3*) relative to wild types. * $P<0.05$ compared to wild type. Error bars denote ± one SEM. *n* and *P* values are detailed in Supplementary Table S3.

Supplementary Figure 2. (**A**) Targeted *sem1* mutants display the characteristic temperature sensitivity phenotype that can be rescued by addition of plasmids expressing wild type *SEM1* or *DSS1* genes (pSEM1 and pDSS1 respectively). Cells are grown to mid-log phase in SC-Trp media (synthetic complete media lacking the amino acid tryptophan) and diluted in water to a starting density of 5,000 cells/μl. Five μl of serial 5-fold dilutions are plated onto control or test plates. Trp is the selectable marker for all plasmids. M101 was isolated from the yeast screen for promoting factors of TNR expansion and has a disruption in the *SEM1* gene. For the temperature sensitivity test, wild type or *sem1* yeast cells containing p*SEM1*, p*DSS1* or the empty vector (pRS314) were spotted on to SC-Trp plates and incubated at 30°C or 37°C. (**B**) The accumulation of polyubiquitinated proteins seen in *sem1* mutants can be rescued by addition of the pSEM1 or pDSS1 plasmids. Cells were grown in SC-Trp media to log phase. Cell extracts were prepared and analysed for polyubiquitinated proteins using an anti-ubqquitin antibody, as described in Materials and Methods. Actin was used as a loading control.

Supplementary Figure 3. Testing drug sensitivity of *sem1* mutants. For all panels cells are grown to mid-log phase and diluted in water to a starting density of 5,000 cells/μl. Five μl of serial 5-fold dilutions are plated onto control or test plates and incubated at 30°C. (**A**) Wild type and *sem1* strains expressing *URA3* were plated on SC-Ura+5-FOA (0.25 g/L) plates to illustrate sensitivity to 5-FOA. The result shows that the *sem1* mutation does not interfere with 5-FOA selection. (**B**) Wild type and *sem1* strains expressing wild type *CAN1* were plated onto SC (control) and SC-Arg+Can (1 μg/ml) plates

and were photographed after 4 days. Canavanine hypersensitivity is evident in *sem1* strains, as previously reported (29). Thus control experiments were performed to ensure that this hypersensitivity does not confound the *CAN1* TNR expansion assay results. (**C**) In a *can1*::*kanMX* background, both wild type and *sem1* strains show resistance to canavanine. This test was performed as described for panel B. We conclude that *sem1* strains are no longer hypersensitive to canavanine when *CAN1* is knocked out, as in our expansion assay strains. (**D**) This experiment tests whether *sem1* and *pre9* mutants with an expanded (CTG) repeat grow similarly to wild type on selective media. These strains have the *can1::kanMX* mutation and contain the *CAN1* $(CTG)_{20}$ reporter integrated at *LYS2* that is His marked. For each strain, a spontaneous expansion was identified that contained circa 30 CTG)repeats, based on PCR analysis (Supplementary Figure S1B). Wild type, *sem1* and *pre9* colonies containing a starting length $(CTG)_{20}$ or an expanded $(CTG)_{30}$ TNR were plated on SC-His (control) or SC-His-Arg+Can (60 μg/ml) plates. The cells were incubated at 30° for 2 days (left panel) or 7 days (right panel). The time, temperature, and selective media are all the same as used when measuring expansion rates. The results indicate that once an expansion has occurred and the *CAN1* gene is no longer expressed the *sem1* and *pre9* strains display similar growth rates as the wild type strain. We conclude that the reduced expansion rates in these mutants cannot be attributed to slow growth on canavanine-containing media.

Supplementary Figure 4. Summary of expansion sizes seen in *sem1*, *pre9* and *sem1 pre9* mutants. (**A**) Histogram displaying the frequency and number

of repeats added for wild type, *sem1*, *pre9* and *sem1 pre9* strains with the $(CTG)_{20}$-*CAN1* reporter as determined by PCR. (**B**) Summary of expansion size data. Weighted mean is calculated as the average of (number of repeats added) x (occurrence). (**C**) Statistical analysis of samples. *P* values were determined by two-tailed Student's *t*-test between the spectra of repeats added.

Supplementary Figure 5. SVG-A TNR expansion assay. (**A**) Schematic of siRNA treatment and TNR expansion assay used in SVG-A cells. Protocols are based on published methods (17,18). Cells are seeded at Day 0 and transfected with siRNA on Day 1. On Day 3 cells are re-transfected with siRNA plus a TNR containing shuttle vector. Cells are harvested on Day 5 and samples are taken for expansion assays, protein or mRNA quantitation, and proteasome activity assays. (**B**) The shuttle vector contains a $(CTG)_{22}$-*CAN1* reporter. Expansions ≥4 repeats, to a final length of 26 or more repeats, block expression of the *CAN1* gene, conferring canavanine resistance (see Supplementary Figure S1A). The shuttle vector contains an SV40 origin that enables plasmid replication in SVG-A cells. The shuttle vector isolated from SVG-A cells post treatment is DpnI digested and transformed into yeast. Yeast is used as a biosensor to score expansions. Total plasmid counts are determined by bacterial transformation for enhanced sensitivity. PCR is used to confirm expansions and analyse expansion sizes. Background levels of expansions are measured by bypassing the SVG-A cells and transforming yeast with stock shuttle vector.

Supplementary Figure 6. Quantification of PSMC5 and PSMB3 protein knockdown for SVG-A experiments. Protein levels were determined by immunoblotting (see Materials and Methods) and analysed using Image J software, normalised to actin loading control and to scrambled siRNA control. Error bars denote ±SEM, *n*=4.

Supplementary Figure 7. Summary of expansion sizes from SVG-A RNAi experiments (**A**) Histogram displaying the frequency and number of repeats added for cells treated with Scrambled (Scr), DSS1, PSMC5 or PSMB3 siRNA. (**B**) Summary of expansion size data.

Supp Fig 1

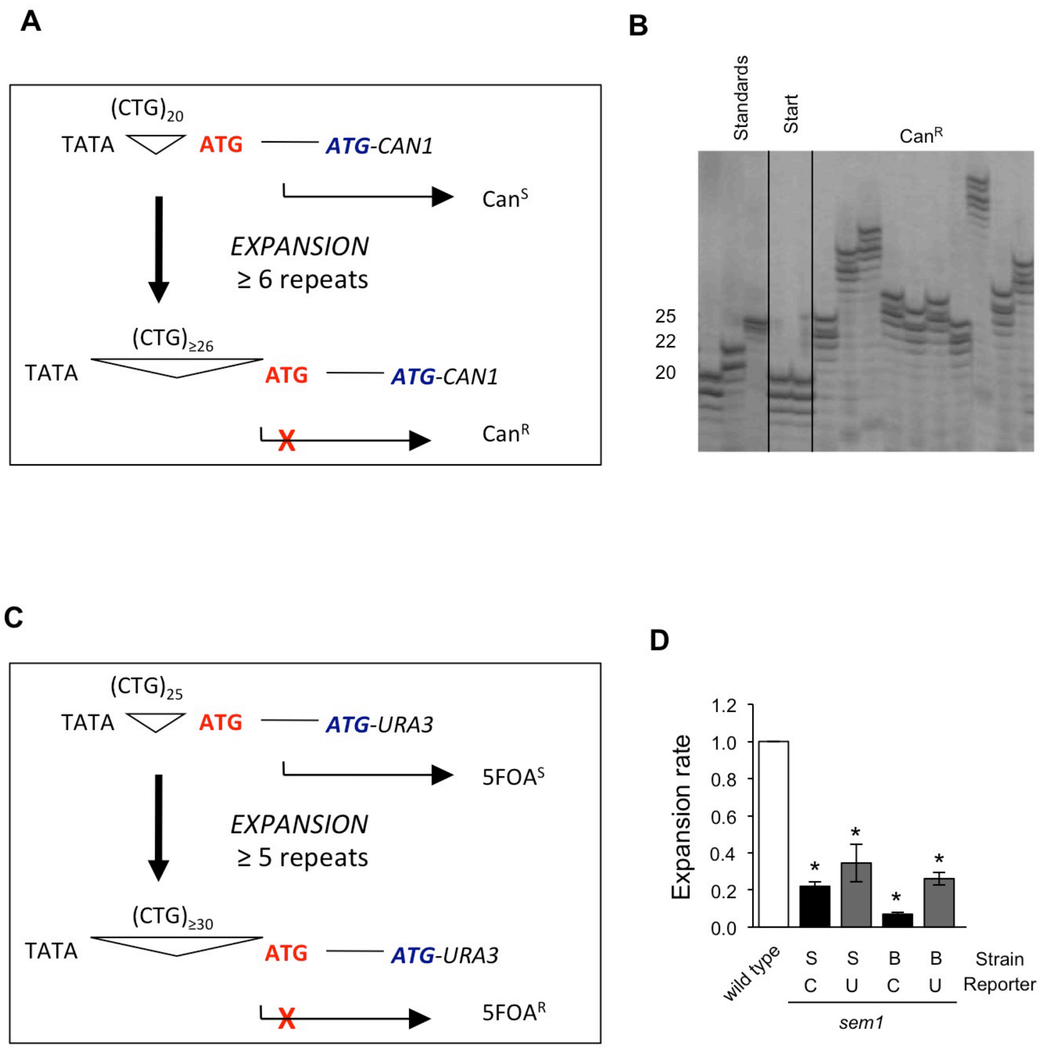
A
(CTG)20
TATA
ATG
ATG-CAN1
CanS
EXPANSION
≥ 6 repeats
(CTG)≥26
TATA
ATG
ATG-CAN1
CanR
B
Standards
Start
CanR
25
22
20
C
(CTG)25
TATA
ATG
ATG-URA3
5FOAS
EXPANSION
≥ 5 repeats
(CTG)≥30
TATA
ATG
ATG-URA3
5FOAR
D
Expansion rate
1.2
1.0
0.8
0.6
0.4
0.2
0.0
wild type
S S B B
C U C U
Strain
Reporter
sem1

Supp Fig 2

A

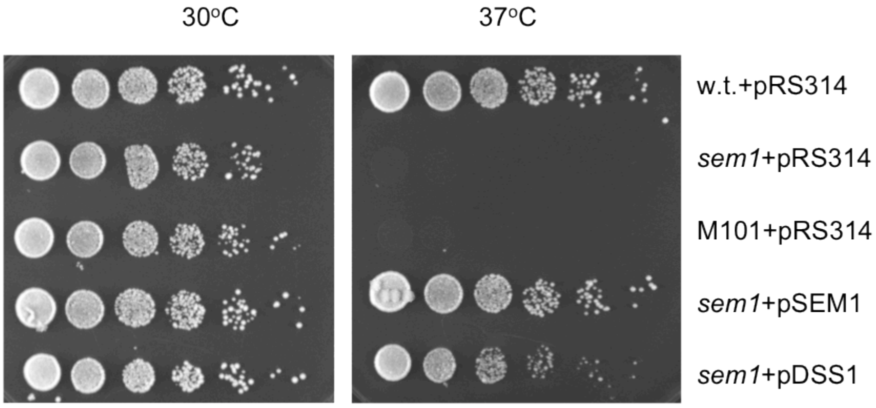
30°C
37°C
w.t.+pRS314
sem1+pRS314
M101+pRS314
sem1+pSEM1
sem1+pDSS1

B

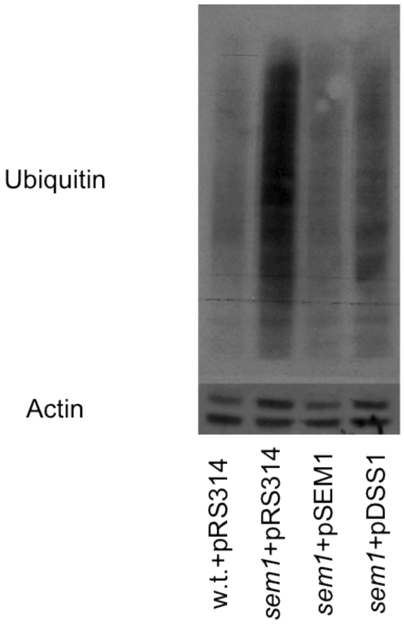
Ubiquitin
Actin
w.t.+pRS314
sem1+pRS314
sem1+pSEM1
sem1+pDSS1

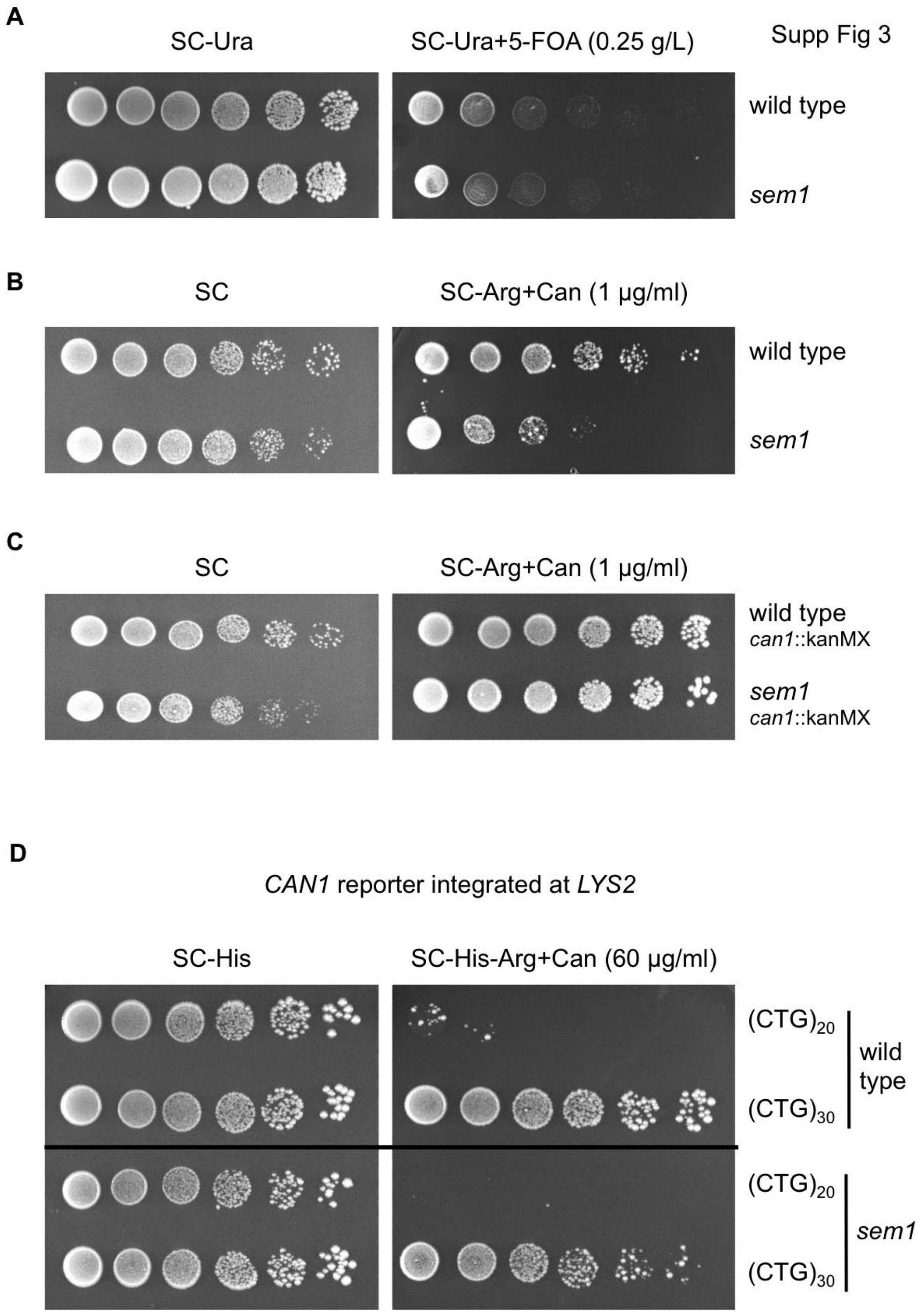
A
SC-Ura
SC-Ura+5-FOA (0.25 g/L)
Supp Fig 3
wild type
sem1
B
SC
SC-Arg+Can (1 µg/ml)
wild type
sem1
C
SC
SC-Arg+Can (1 µg/ml)
wild type
can1::kanMX
sem1
can1::kanMX
D
CAN1 reporter integrated at *LYS2*
SC-His
SC-His-Arg+Can (60 µg/ml)
$(CTG)_{20}$
$(CTG)_{30}$
wild type
$(CTG)_{20}$
$(CTG)_{30}$
sem1

Supp Fig 4

A

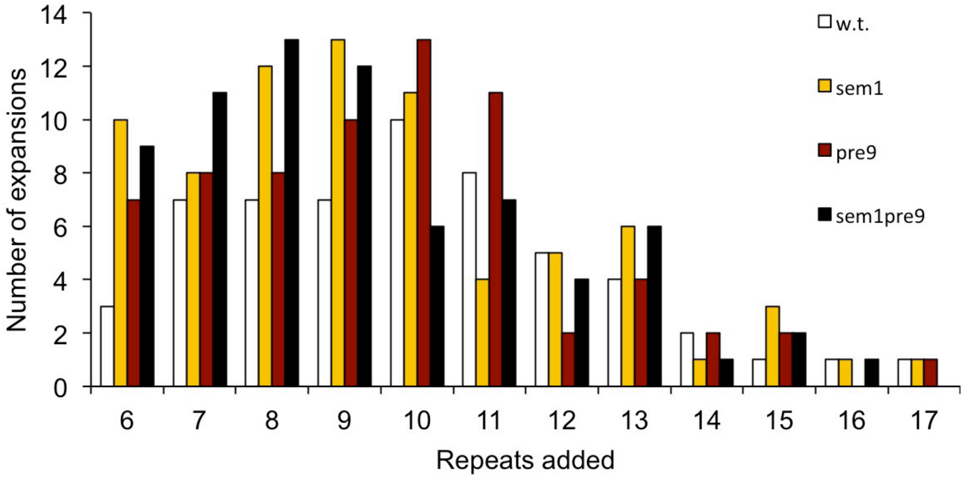

B

Strain	n	range	weighted mean	median
wild type	56	6 to 17	10.04	10
sem1	75	6 to 17	9.51	9
pre9	68	6 to 17	9.62	10
sem1 pre9	72	6 to 15	9.25	8

C

P **values**	**vs w.t.**	**vs *sem1***	**vs *pre9***
w.t.	-	0.26	0.23
sem1	0.26	-	0.80
pre9	0.23	0.80	-
sem1 pre9	0.08	0.55	0.38

Supp Fig 5

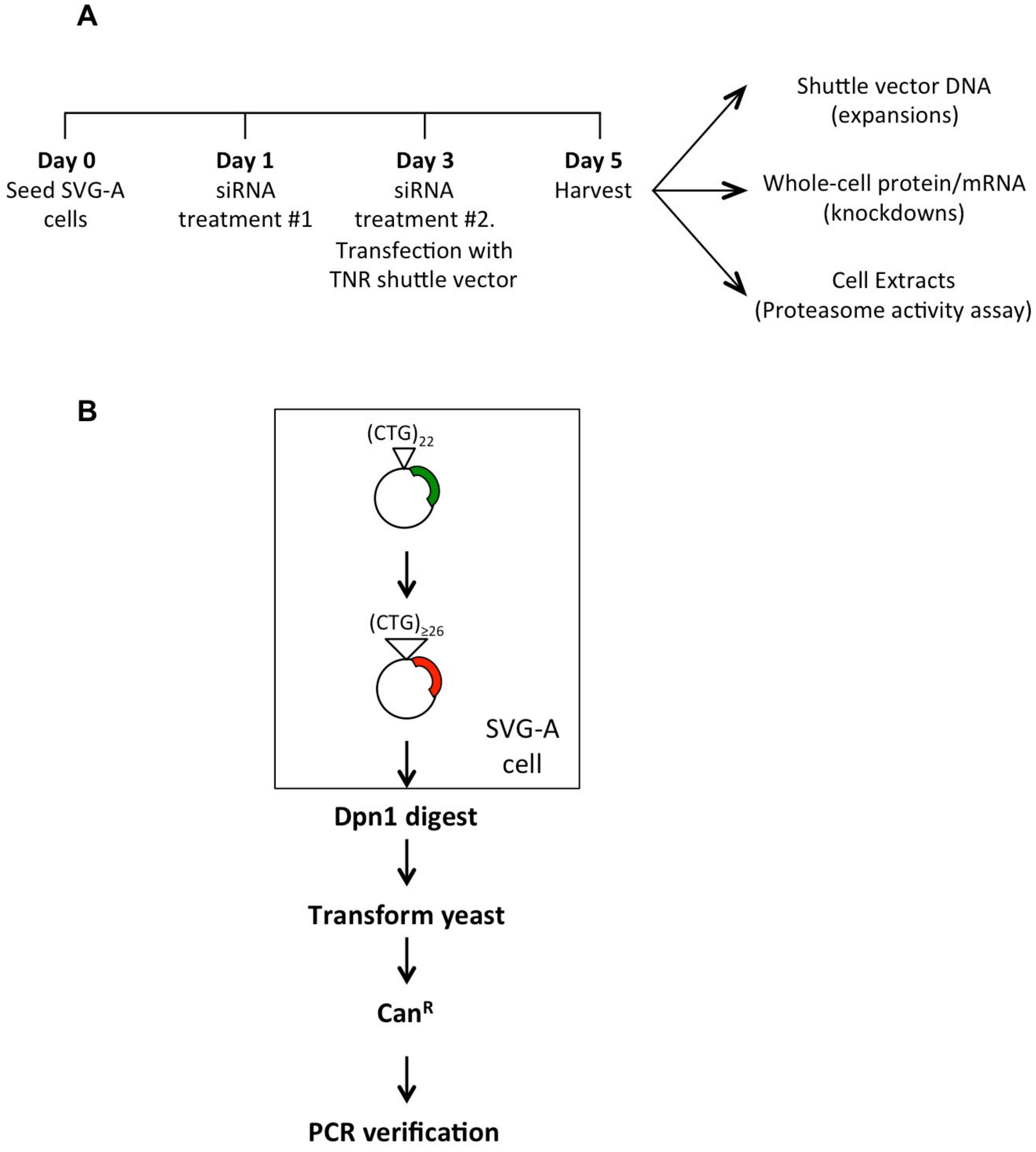
A
Day 0
Seed SVG-A cells
Day 1
siRNA treatment #1
Day 3
siRNA treatment #2. Transfection with TNR shuttle vector
Day 5
Harvest
Shuttle vector DNA (expansions)
Whole-cell protein/mRNA (knockdowns)
Cell Extracts (Proteasome activity assay)
B
(CTG)22
(CTG)≥26
SVG-A cell
Dpn1 digest
Transform yeast
CanR
PCR verification

Supp Fig 6

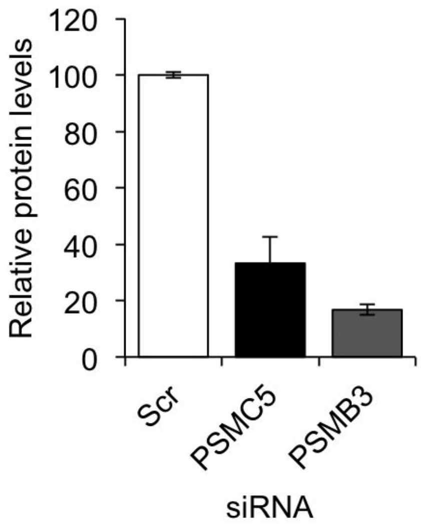

Relative protein levels
120
100
80
60
40
20
0
Scr
PSMC5
PSMB3
siRNA

Supp Fig 7

A

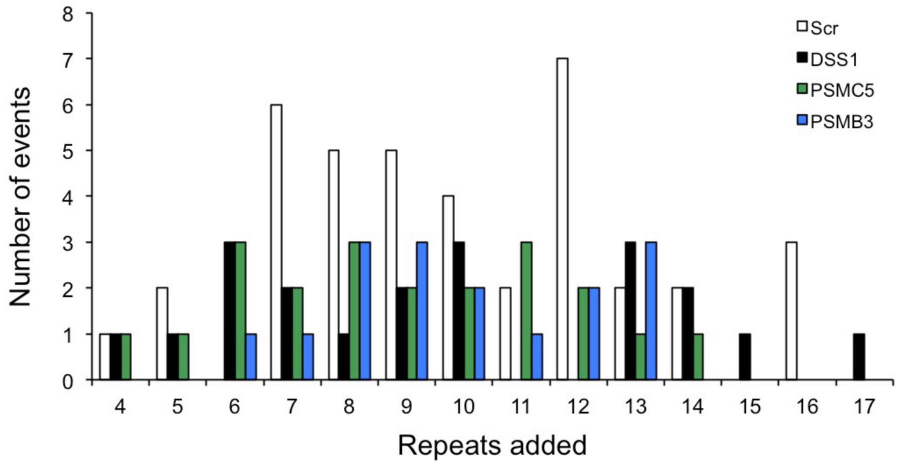

B

RNAi	n	range	weighted mean	median	*P* vs Scr
Scr	34	4 to 16	10	12	
DSS1	32	4 to 17	9.8	10	0.66
PSMC5	30	4 to 14	8.9	8	0.19
PSMB3	31	6 to 12	9.9	9	0.78
